# Seroprevalence of hepatitis B virus infection and associated factors among healthcare workers in northern Tanzania

**DOI:** 10.1186/s12879-018-3376-2

**Published:** 2018-09-21

**Authors:** Elichilia R. Shao, Innocent B. Mboya, Daniel W. Gunda, Flora G. Ruhangisa, Elizabeth M. Temu, Mercy L. Nkwama, Jeremia J. Pyuza, Kajiru G. Kilonzo, Furaha S. Lyamuya, Venance P. Maro

**Affiliations:** 1 Internal Medicine Department, Kilimanjaro Christian Medical Center, P.O.Box3010, Moshi, United Republic of Tanzania; 2 Institute of Public Health, Community Health Department, Kilimanjaro Christian Medical University College, P.O.Box2240, Moshi, Tanzania; 3 CUHAS, P.O.Box 1370, Mwanza, Tanzania; 4 Better Human Health Foundation, P.O.Box1348, Moshi, Tanzania; 5 Imagedoctors International, P.O.Box16341, Arusha, Tanzania

**Keywords:** Hepatitis B virus, Risk factors, Healthcare workers, Tanzania

## Abstract

**Background:**

Hepatitis B virus infection is a global health problem with the highest prevalence in East Asia and Sub-Saharan Africa. The majority of infected people, including healthcare workers are unaware of their status. This study is aimed to determining seroprevalence of hepatitis B virus infection and associated factors among healthcare workers in northern Tanzania.

**Methods:**

This cross-sectional study included 442 healthcare workers (HCWs) from a tertiary and teaching hospital in Tanzania before the nationwide hepatitis B vaccination campaign in 2004. Questionnaire- based interviews were used to obtain detailed histories of the following: demographic characteristics; occupation risks such splash and needle stick injuries or other invasive procedure such as intravenous, intramuscular or subcutaneous injections; history of blood transfusion and surgeries, as well as HCWs’knowledge of HBV. Serological markers of HBV were done using Laborex HBsAg rapid test. Serology was done at zero months and repeated after six months (bioscienceinternational.co.ke/rapid-test-laborex.html HBsAg Piazzale-milano-2, Italy [Accessed on November 2017]). Chi-square (χ^2^) tests were used to compare proportion of HBV infection by different HCWs characteristics. Multivariable logistic regression was used to determine factors associated with HBV infection.

**Results:**

A total of 450 surveys were sent out, with a 98.2% response rate. Among the 442 HCWs who answered the questionnaire, the prevalence of chronic hepatitis B virus infection was 5.7% (25/442). Only 50 (11.3%) of HCWs were aware of the HBV status. During the second HBsAg testing which was done after six months one participant sero-converted hence was excluded. Adjusted for other factors, history of blood transfusion significantly increased the odds of HBV infection (OR = 21.44, 95%CI 6.05, 76.01, *p* < 0.001) while HBV vaccine uptake was protective against HBV infection (OR = 0.06, 95%CI 0.02, 0.26, p < 0.001). The majority of HCWs with chronic HBV infection had poor to fare knowledge about HBV infection but this was not statistically significant when controlled for confounding.

**Conclusions:**

Prevalence of HBV among health care workers was 5.7% which is similar to national prevalence. Although the response rate to take part in the study was good but knowledge on HBV infection among HCWs was unsatisfactory. History of blood transfusion increased risks while vaccine uptake decreased the risk of HBV infection. This study recommends continues vaccinating HCWs together with continues medical education all over the country. We also recommend documentation of vaccination evidence should be asked before employment of HCWs in order to sensitize more uptakes of vaccinations. Although we didn’t assess the use of personal protective equipment but we encourage HCWs to abide strictly on universal protections against nosocomial infections.

## Background

About 240 million people are living with chronic hepatitis B virus (HBV) infection worldwide, majority of them from low-and middle income countries. HBV is a major causative agent of chronic hepatitis and can cause liver cirrhosis and hepatocellular carcinoma [1, 2]. Most people including health care workers (HCWs) are unaware of their HBV serological statues. Antiretroviral drugs like Entecavir and tenofovir reduces aggressiveness of the disease and reduces mortality [3–5]. However, due to unawareness of HBV status in most of developing countries, implementation of treatment strategies has not been well established. HCWs have an increased risk of hepatitis B infection about four times as compared to general population because of their direct contact with infectious material such as blood, needle stick injuries and other contaminated body fluids [6]. Prevalence of hepatitis B infection among general population in Tanzania is 6%; HCWs are at increased risk to infection because of their working environment [7–10].About 3 million HCWs globally, experience percutaneous exposure to blood pathogens each year. Those who perform invasive procedures such as surgeons, emergency medicine expertise, laboratory personnel and pathologists shown to have higher prevalence as compared to others specialities. The different in prevalence can be explained by the different degree of exposures to the risks [11].Other factors which might increase the risks to contract HBV includes medical procedures such as surgeries, blood and blood products transfusions, endoscopic procedures and dialysis. Sexual behaviour such as unprotected sex with multiple partners in endemic area is among important an aspect which is associated with increased risks to HBV infection [12].

Risk factors to contract HBV have been studied by different authors basing on the questions such as HBV is presents in high concentration in blood fluids. Hepatitis B virus infection is more infectious than HIV, can cause liver cancer; can stay alive out of blood for seven days [13]. Factors such as unprotected sexual intercourse, blood transfusion, needle stick injuries, splash, intravenous and/or intramuscular injections, and unvaccinated population has been associated with increased risks toward hepatitis B infections [14, 15]. Knowledge about HBV infection has a big impact on a person’s decision toward preventive measures hence can influence sero-positivity. There is growing body of literature in Tanzania on HBV infection but there limited information on associated risk factors among HCWs. This article contributes information on the seroprevalence and associated risk factors towards HBV infection among HCWs.

## Methods

### Setting and study population

This study was conducted among HCWs from January 2015 to December 2016 at Kilimanjaro Christian Medical Centre (KCMC) in Moshi, Tanzania. KCMC is a referral and teaching hospital which serve over 15 million people in Northern Tanzania. It is a huge complex facility with 800 inpatients, 1852 students and 1300 employees [16]. Study design, sampling and participant’s recruitmentA total of 442 HCWs aged 18 years and above participated in this study; making a response rate of 98.2%.Enrolled was done in two categories, those from surgical and none surgical departments. Our research team had one group dealing with workers at night shift and the other group with day shift. Category A: Surgical departments (that included Obstetrics and Gynaecology, General surgery, Orthopaedic surgery, Eye Nose and Throat, Urology and Eye department). Category B: None-surgical department (that included medical, paediatrics, dermatology, emergency/OPD, Orthopaedic workshop, as well as laboratory and administration). After consent, HCWs participated in this study were voluntary enrolled consecutively until desired number was met. Consenting participants were asked to fill a self-administered questionnaire after they had provided a blood sample. Unique identification number was used to link participant’s laboratory results and the questionnaire. The standardized questionnaire was used to collect information like demographic data, years at work, medical history, education level and professional. The questionnaire had four sections: demographic and academic characteristics, knowledge of the risk factors for HBV infection and vaccine and history of accidental exposure to blood and its products. A detailed history was obtained from the HCW on hepatitis B vaccination and infection history, any occupation risks such (splash and needle stick injuries). Other invasive procedure such as Intravenous (IV), Intramuscular (IM), subcutaneous (SC) injections, blood transfusion history, surgery and all these findings were all recorded. Other risk behaviours like multiple sexual partners without using protection were also asked from the participants. Knowledge measurements were done by adopting an approach used by Abdul-Hakeem et al. 2016. In this study, each correct response “answered Yes”) to the 16 questions on knowledge on HBV infection and vaccination was scored one mark while none or don’t know was scored zero. The total scores were converted to percentage and grouped as poor (< 50%), fair (50–74%) and good (≥75%) [17].

### Ethical issues

Ethically this study was approved by KCMUCo Research and Ethics Review Committee with permission number 916. All participants signed an informed consent. Counselling and blood collection were done at the private room and no names was used instead identification numbers was used. All questionnaires were stored in the secure room at KCMC while data for this study were secured in a password protected laptop. Those who tested positive for HBsAg were counselled and channelled to the physician for further evaluation. Benefit for the study participantTo date no antiviral treatment is licensed for treating chronic HBV infection in Tanzania. But knowing your hepatitis status is a step towards monitoring and treatment. We invited family members and sexual partners of infected participants for testing and further assessment.

### Serological analysis for HBV

Under aseptic technique about (4 mL) of blood was obtained through venipuncture from all subjects using sterilized disposable 5 ml syringes and 20 gauge needles by laboratory technologist who is experienced phlebotomist at KCMC.All collected blood samples were tested using Laborex HBsAg rapid test [18], Milan Italy for positive hepatitis B surface antigen. The test strip was removed from the foil pouch and dipped into the specimen for at least 10 s until thoroughly wet. Then the strip was removed from the specimen and placed in the dry flat surfaces to wait for the red lines to appear. Interpretation of the results: Positive; when two distinct red lines appears, one line in the control region (C) and another one in the test region (T).Negative: One red line appears in the control (C). No apparent red or pink line appears in the test region (T). Invalid: Control line fails to appear. Quality control: appearance of red line in the control region (C) was the internal procedural control. It confirms sufficient specimen volume and correct procedure technique. For those which turned to be positive participants were counselled and informed to come and repeat the test after six months. Those who still turned positive after six months were termed chronic hepatitis B infected HCWs. All the methods used were proved by laboratory standard in Tanzania.

### Statistical analysis

Data were entered in Microsoft excel and analysis was performed using STATA version 13.1. Descriptive analysis was performed whereby numeric variables were summarized using measures of central tendency and the corresponding measures of dispersion. Categorical variables were summarized using frequency and percentages. Chi-square (χ2) test was used to compare the prevalence of hepatitis B infection by participant characteristics. Multivariable logistic regression (Odds ratio and the corresponding 95% confidence interval) was used to determine factors associated with hepatitis B infection among health care workers at KCMC. A cut-off point of 10% (*p* > 0.1) was used to select variables to be included in further analysis. After controlling for potential confounders, variables with *p* < 0.05 was considered statistically associated with hepatitis B infection.

## Results

### Patient characteristics

A total of 442 health workers was included in this study. The median age of participants was 37 years and interquartile range (IQR) of 31–46 years. About 189 (43%) were aged above 40 years while 165 (37.3%) were aged between 30 and 39 years. Over 270 (60%) of all participants were females and above three quarter 344 (78.5%) had tertiary education level. Also 275 (62%) of all participants were married or cohabiting 378 (85.7%) The majority of the participants were from none-surgical 248(56.1%) departments (Table 1). Majority of HCWs who were seropositive for HBsAg were mainly medical doctors 6(24%), nurses 5(20%) followed by laboratory personnel 4(16%) and ward attendants 4(16%).

**Table 1:** Demographic characteristics of HCWs at KCMC Moshi Tanzania 2016/17 (*N* = 442)

Variable	Frequency (n)	Percentage (%)
Age (years)		
Median (IQR)	37	(31, 46)
20–29	88	19.9
30–39	165	37.3
40+	189	42.8
Sex		
Male	166	37.6
Female	276	62.4
Education level		
Primary	27	6.2
Secondary	67	15.3
Tertiary	344	78.5
Marital status		
Single	140	31.7
Married/Cohabiting	275	62.2
Widow/Divorced	27	6.1
Religion*		
Christian	378	85.7
Muslim	63	14.3
Area of residence		
Rural	187	42.3
Urban	255	57.7
Department		
Surgical	194	43.9
Non-surgical	129	29.2
Outpatient/Emergency	21	4.8
Administrative/Supportive	42	9.5
Laboratory/Pathology	56	12.7
Specialty		
Doctor	130	29.4
Nurse	137	31.0
Laboratory	37	8.4
Other health professions	56	12.7
Administrative/ Supportive staff	82	18.6
Work in clinical area		
Yes	350	79.2
No	92	20.8
Years of practice*		
< 5	91	21.0
≥5	342	79.0

*Frequency do not tally to the total due to missing information on religion

### Exposure to the risk of HBV infection

Among all participants in this study, 145 (32.8%) had history of surgery, 121 (27.4%) had history of blood splash to the eyes or mouth, 367 (83.0%) every had intravenous injections, 387 (87.6%) had intramuscular injections, 167 (37.9%) had a history of needle stick injury, less than 10% had history of invasive procedure such as endoscopy and about 8% ever had blood transfusion. Prevalence of HBV vaccine uptake in this study was 67.4% (Table 2).

**Table 2:** Exposure to the risk of HBV infection among healthcare workers at KCMC Moshi Tanzania 2016/17

Variable	n	%
History of surgery		
Yes	145	32.8
No	297	67.2
History of blood splash to the eyes or mouth		
Yes	121	27.4
No	319	72.2
Don’t know	2	0.5
Ever had intravenous injections		
Yes	367	83.0
No	75	17.0
Ever had intramuscular injections		
Yes	387	87.6
No	55	12.4
History of needle stick injury*		
Yes	167	37.9
No	274	62.1
History of invasive procedure such as endoscopy*		
Yes	40	9.1
No	399	90.5
Don’t know	2	0.5
Ever had blood transfusion		
Yes	34	7.9
No	399	92.2
HBV Vaccine uptake*		
Yes	295	67.4
No	143	32.6

*Frequency do not tally to the total due to missing values

### Prevalence of HBV

Prevalence of chronic HBV infection (HBsAg) was reported to be 5.7% (25/442). During the second testing which was done after six months one participant sero-converted hence was excluded. There was no significant difference in contracting HBV between males and females (χ2 = 0.432; *p* = 0.51). Majority of seropositive HCWs were > =30 yrs. but the difference was not significant (*p* = 0.93). Years of practices were not associated with seropositivity (*p* = 0.13), Table 3.

**Table 3:** Demographic characteristics associated with Hepatitis B virus infection among HCWs at KCMC Moshi, Tanzania 2016/17

Variable	Total	Serology n (%)		χ^2*^	p-value
		Negative	Positive		
Age (years)				0.278	0.93**
20–29	88	84 (95.4)	4 (4.6)		
30–39	164	154 (93.9)	10 (6.1)		
40+	187	176 (94.1)	11 (5.9)		
Sex				0.432	0.51
Male	166	155 (93.4)	11 (6.6)		
Female	273	259 (94.9)	14 (5.1)		
Education level				1.835	0.30**
Primary	26	24 (92.3)	2 (7.7)		
Secondary	67	61 (91.0)	6 (9.0)		
Tertiary	342	325 (95.0)	17 (5.0)		
Marital status				4.212	0.14**
Single	140	128 (91.4)	12 (8.6)		
Married/Cohabiting	272	259 (95.2)	13 (4.8)		
Widow/Divorced	27	27 (100)	0 (0)		
Religion				3.992	0.05
Christian	375	357 (95.2)	18 (4.8)		
Muslim	63	56 (88.9)	7 (11.1)		
Area of residence				1.001	0.32
Rural	186	173 (93.0)	13 (7.0)		
Urban	253	241 (95.3)	12 (4.7)		
Department				2.653	0.47**
Surgical	193	183 (94.8)	10 (5.2)		
Non-surgical	129	122 (96.1)	5 (3.9)		
Outpatient/Emergency	21	19 (90.5)	2 (9.5)		
Administrative/Supportive	42	39 (92.9)	3 (7.1)		
Laboratory/Pathology	56	51 (91.1)	5 (8.9)		
Specialty				3.076	0.48**
Doctor	130	123 (94.6)	7 (5.4)		
Nurse	135	130 (96.3)	5 (3.7)		
Laboratory	37	33 (89.2)	4 (10.8)		
Other health professions	56	52 (92.9)	5 (7.1)		
Administrative/ Supportive staff	81	76 (93.8)	5 (6.2)		
Work in clinical area				1.229	0.32**
Yes	348	326 (93.7)	22 (6.3)		
No	91	88 (96.7)	3 (3.3)		
Years of practice				2.756	0.13**
< 5	91	89 (97.8)	2 (2.2)		
≥5	339	316 (93.2)	23 (6.8)		

*Chi-square; **p-value by Fisher’s exact estimation

Sixty percent of chronic HBV infection was found among none-surgical HCWs with no statistical significance difference between these groups (*p* = 0.47). We found that history of blood transfusion and surgery were associated with seropositivity (*p*-value = 0.001 and 0.003) respectively. Likewise, history of vaccine uptake was also associated with HBV infection (*p* < 0.001), Table 4. Among HCWs who were positive for HBV, 6(20%) were medical doctors; two from none-surgical department and the rest were from surgical department (Tables 4).

**Table 4:** HBV infection by past exposure to potential risk factors among HCWs at KCMC Moshi Tanzania 2016/17

Variable	Total	Serology n (%)		χ^2*^	p-value
		Negative	Positive		
History of surgery*				8.897	0.003**
Yes	144	129 (89.6)	15 (10.4)		
No	295	285 (96.6)	10 (3.4)		
History of blood splash to the eyes or mouth*				2.137	2.26**
Yes	121	111 (91.7)	10 (8.3)		
No	316	301 (95.3)	15 (4.7)		
Don’t know	2	2 (100)	0 (0)		
Ever had intravenous injections*				0.446	0.78**
Yes	365	343 (94.0)	22 (6.0)		
No	74	71 (96.0)	3 (4.0)		
Ever had intramuscular injections*				0.414	0.75**
Yes	386	363 (94.0)	23 (6.0)		
No	53	51 (96.2)	2 (3.8)		
History of needle stick injury*				0.452	0.50
Yes	165	154 (93.3)	11 (6.8)		
No	273	259 (94.9)	14 (5.1)		
History of invasive procedure such as endoscopy*				0.151	1.00**
Yes	39	37 (94.9)	2 (5.1)		
No	397	374 (94.2)	23 (5.8)		
Don’t know	2	2 (100)	0 (0)		
Ever had blood transfusion*				65.946	< 0.001
Yes	34	22 (64.7)	12 (35.3)		
No	399	388 (97.2)	11 (2.8)		
HBV Vaccine uptake*				23.420	< 0.001**
Yes	293	289 (98.6)	4 (1.4)		
No	142	125 (88.0)	17 (12.0)		
Knowledge on HBV infection				5.886	0.04**
Poor	108	98 (90.7)	10 (9.3)		
Fair	217	204 (94.0)	13 (6.0)		
Good	114	112 (98.2)	2 (1.8)		

*Frequencies do not tally to the total due to missing values either in the outcome variable (serology status) or the independent variables, this also causes variations from numbers in Table 4; **p-value by Fisher’s exact estimation

### Knowledge on HBV infection

The median knowledge score was 62.5% and interquartile range of 50–75%. Nearly a quarter (25.4%) of all participants had good knowledge and about half (49.6%) had fair knowledge about HBV infection (Table 5). Only 17.9% of participants were aware, that unprotected sex with multiple partners was the risks for HBV infection. Most of the participants (85.9%) correctly identified that HBV is more contagious than HIV, while (91.3%) knew that there is effective and safe hepatitis B vaccine. About (23.4%) of HCWs wrongly understood that you can get HBV infection from DNA-recombinant vaccine.

**Table 5:** Knowledge on HBV infection and vaccination among healthcare workers at KCMC* Moshi Tanzania 2016/17 (N = 442)

Statement	n	%
1. HBV can be transmitted through sexual intercourse	354	80.6
2. HBV can be transmitted through unprotected sex with multiple sexual partner	79	17.9
3. HBV is the most contagious blood-borne pathogen through accidental exposure to blood and its products	379	85.9
4. Injury with needle contaminated with infected blood is the risk factor of HBV infection	391	89.1
5. Contact with broken skin with infected body fluid is a risk factor of HBV infection	365	83.0
6. Contact to mucous membrane in the eyes or mouth with infected blood is a risk factor of HBV infection	321	72.8
7. Contact of healthy skin with infected blood or products is a risk factor of HBV infection	144	32.7
8. HBV infection can be transmitted through oral-fecal route	134	30.4
9. HBV could be transmitted from a mother to her fetus	290	65.9
10. Immunoglobulin against HBV can prevent infection after exposure	233	53.0
11. There is a vaccine which is available against HBV	398	91.3
12. If Hepatitis B vaccination is taken properly as per protocol, it is more than 95% protective against HBV infection	340	77.3
13. The minimum numbers of doses for a complete primary HBV vaccination is three doses	278	63.0
14. An immune response test should be done after HBV vaccination	199	46.5
15. Antibody titer above 10 IU is the recommended amount which is protective	138	31.3
16. You can get HBV infection from recombinant HBV vaccination	103	23.4
Knowledge level		
Median (IQR)	62.5	(50, 75)
Poor	109	24.7
Fair	219	49.6
Good	114	25.8

*Frequency and percentage distributions of only those who answered correctly in these statements

### Factors associated with HBV

Several factors showed significant association with HBV seropositivity in the crude analysis. This includes history of surgery (OR = 3.31, 95%CI 1.44, 7.58), history of blood transfusion (OR = 19.24, 95%CI 7.64, 48.47) and having good knowledge on HBV infection (OR = 0.18, 95%CI 0.04, 0.82) compared to those with poor knowledge (Table 6). The final model was adjusted for religion, history of surgery, history of blood transfusion, HBV vaccine uptake and HBV knowledge. History of blood transfusion and HBV vaccine uptake were the only factors associated with HBV infection (Table 6). HCWs with history of blood transfusion had over 21 times the higher odds of HBV infection compared to those without transfusion (OR = 21.44, 95%CI 6.05, 76.01) while those with history of vaccine uptake were protected against HBV infection (OR = 0.06, 95%CI 0.02, 0.26).

**Table 6:** Univariate and multivariate logistic regression for factors associated with HBV infection among HCWs at KCMC Moshi, Tanzania 2016/17

Variable	Total	+Ve serology n (%)	cOR* (95%CI)	*p*-value	aOR** (95%CI)	*p*-value
Religion						
Muslim	63	7 (11.1)	Reference		Reference	
Christian	375	18 (4.8)	0.40 (0.16, 1.01)	0.05	1.05 (0.26, 4.18)	0.94
History of surgery						
No	295	10 (3.4)	Reference		Reference	
Yes	144	15 (10.4)	3.31 (1.44, 7.58)	0.005	2.31 (0.77, 6.90)	0.13
Ever had blood transfusion						
No	399	11 (2.8)	Reference		Reference	
Yes	34	12 (35.3)	19.24 (7.64, 48.47)	< 0.001	21.44 (6.05, 76.01)	< 0.001
HBV Vaccine uptake*						
No	142	17 (12.0)	Reference		Reference	
Yes	293	4 (1.4)	0.10 (0.03, 0.31)	< 0.001	0.06 (0.02, 0.26)	< 0.001
Knowledge on HBV infection						
Poor	108	10 (9.3)	Reference		Reference	
Fair	217	13 (6.0)	0.62 (0.26, 1.47)	0.28	0.48 (0.14, 1.65)	0.25
Good	114	2 (1.8)	0.18 (0.04, 0.82)	0.03	0.38 (0.07, 2.09)	0.25

*Crude odds ratio; **Adjusted odds ratio

## Discussion

This study was carried out to determine seroprevalence of chronic HBV infection and risk factors associated with seropositivity among HCWs in a Tanzanian tertiary and teaching hospital. Serological testing revealed that 5.7% of HCWs were chronic carriers while 94.3% were susceptible to HBV infection. These workers were infected with hepatitis B virus were counselled and given feedbacks to do a further workout and channelled to the respective clinics. The response rate of 98.2% out of 450 study participants was achieved because of the good relationship between participants and research team. There were two groups of research team, night team dealing with those workers at night shift and day team for day shift. These HCWs might transmit HBV infection to the patients as well as their family members. We counselled them to bring their sexual partners and children to be tested for HBV infection. A similar study conducted in Mwanza among HCWs showed the prevalence of 7%, but was almost twice the prevalence among pregnant women, and less than pregnant women who were HIV positive and blood donors. The slight different in this study as compared to the previous study by Mueller et al. might be explained by the methodological components. The recent mini review article by Kilonzo et al., presented the national prevalence of HBsAg among general population to be 6% which is similar to our study [7, 15–17, 19–22]. In this study we only did HBsAg and not anti-HBc + which might tell us the HCWs who cleared the infection [9].

In a similar study in the neighbouring country, Uganda showed the prevalence of 8.1%. Other studies of HBsAg among HCWs reported the prevalence ranging from 1.5–8.1% in Nigeria, Rwanda and Uganda. Nigeria is among the countries with high prevalence in the general population when compared to Tanzania. Furthermore in the study which was done by Abiola et al., the prevalence of HBsAg was as low as 1.5% which might be explained by the low sample size of participants as compared to our study [23–26]. Majority of seropositive HCWs in this study were medical doctors, nurses followed by laboratory personel. This was different from Uganda study by Ziraba et al., who showed that nursing assistant was leading followed by laboratory technicians [6]. The difference might be explained by the different levels of risk of exposure to potential risks environment. Out of six medical doctors who were seropositive for HBV, 66.7% were from none surgical-department as compared to the rest who were from the surgical departments. Different level of potential risks exposure might affect the prevalence of the particular nosocomial infection. However, we asked about the sexual history, but it was prone to bias because the researchers were from the same institutions. But we believe it was controlled in one way or another because no names were used instead identification number was used. Other factors such as ethnicity, region of origin, years of practices and religion might influence the chance of HCWs to be infected or not. In the current study we could not compare the seroprevalence among different religious groups because of unequal distribution of this group [27].

In this study we found greater than 19-fold higher risk for HCWs to acquire HBV infection if they ever had the blood transfusion (odds ratio 19.24, p < =0.001). This might be explained by the methodology used for screening as well as the incubation period of the disease. Furthermore the occult HBV infection can explain this because for the donated blood is always screened by doing HBsAg and hepatitis core-antibody but studies have revealed an HBV DNA positivity rate of 0–15% when HBV nucleic acid testing (NAT) was employed [20].Therefore in order to reduce transfusion transmissible infection we might opt for HBV nucleic acid instead of our traditional methods which has some challenges due to the pathogenesis of HBV. The study found higher rate of HBV infection in older workers as compared to younger ones. This could be explained by more exposure during life time to among older HCWs or less exposure among younger HCWs.Also, we learned that majority about 92% of seropositive HCWs have more than five years working experience which support the assumption of more exposure. Our findings are similar to the study by Mueller et al., in Lake Zone Mwanza Tanzania [8]. Many years in practice means increased occupational exposure in clinical services which increases the chances of acquiring HBV infection, this in similar in other studies [6, 26].

This study was conducted before nationwide vaccination campaign against all HCWs which was started January 2015. Therefore, the vaccination coverage was 19.6% which was similar with the one reported in Mwanza study among HCWs. We could not get complete information about the number of doses and if they did screening before vaccination. These vaccinations were done among HCWs who were involved in different research projects runned by KCMC in collaboration with international agents. This proportion seems to be higher as compared to other studies in SSA [8, 26, 27]. There was association between vaccination uptake and seropositive of HBV infection. After adjustment for the confounding effect, HBV vaccine uptake remained to be statistically significant. Although the vaccination coverage in this study was very low when compared to developed countries in Europe [28, 29].In resource limited settings like Tanzania, most HBV infection take place during childhood due to high endemicity nature of hepatitis B virus infection. This has been supported by our study which didn’t show any association between age and years of practices, which means many HCWs, has been infected during childhood rather adulthood as occupational hazards. HCWs can be infected via occupational exposure to infected clients or vice versa. Therefore, vaccination is still the main soul for the control of new infections in this region.

The percentage of good knowledge was very low as compared to the studies in Nigeria which was 69% [19]. About half of the participants didn’t know that antibody titer should be done after finishing the third dose. This was similar to other studies in Cameroon where about one-fourth of the HCWs had good knowledge about hepatitis B infection and vaccination [15]. Good level of knowledge was protective towards hepatitis B infection, therefore those with poor knowledge has more chances of been infected. After adjusting for other confounders there was no statistical significant between knowledge level and seropositivity for HBV. We had about 20% of HCWs who responded that hepatitis B cannot be transmitted through unprotected sexual intercourse. About quarter of the participants responded that HBV cannot be transmitted through contact with mucous membrane in the eyes and mouth. Furthermore 30 % of participants wrongly perceived that hepatitis B can be transmitted through feaco-oral route. It seems that many HCWs are not knowledgeable about many aspects of HBV from its pathogenesis, transmission as well as preventive measures. The percentage of poor knowledge is this study was higher than the study in Northwest Ethiopia [11]. One third of the workers didn’t understand that HBV can be transmitted from mother to child; surprisingly 10% of the HCWs had no information about the availability of effective and safe hepatitis B vaccine. More than 50 % of HCWs didn’t understand that they needed to do antibody titters after finishing three doses of hepatitis B vaccination. Quarter of the workers responded that you could get hepatitis B infection from the DNA recombinant Hepatitis B vaccine. This is less than the finding from South Korea where about 56% of family medicine residence declined to take hepatitis B infection because of the fear that they might develop infection from the given recombinant vaccine [5, 25]. Continues medical education is very crucial for HCWs, but vaccination against HBV infection remain the most effective and safe method toward elimination of hepatitis B infection.

## Limitations of the study

We only did HBsAg therefore we cannot conclude about the number of HCWs exposed to hepatitis B virus which can be done by HBsAb. Since we were dealing with our colleagues, some questions might be too personal, hence respond, will have bias. Also, most of the information given by HCWs was prone to recall bias due to time interval between the event and the time we asked questions. Questions like unprotected sex with multiple sexual partners faced some challenges where some HCWs didn’t respond.

## Conclusions

The prevalence of hepatitis B virus infection among healthcare workers was high. Majority of HCWs were exposed to potential risks of infection but only small percentages were vaccinated against HBV. These findings highlight the need for continues improving the working environment of HCWS by provision of hepatitis B vaccination. Continuous medical education to HCWs should be reinforced to improve their knowledge in order to protect them. Vaccination program to HCWs should continue to be implemented either mandatory or optional framework. It is the right time to expand this study to involve other health facilities in order to be able to formulate evidenced national data for action. Although we didn’t assess the use of personal protective equipment but we encourage strictly abide on universal protections against nosocomial infections.

## Abbreviations

AIDS: Acquired Immune Deficiency Syndrome
ALT: Alanine aminotrasnferase
Ant-HBC: Antibody to Hepatitis B core protein
Ant-HBe: Antibody to Hepatitis B e antigen
Anti HBC IgM: Hepatitis B core protein antibody immunoglobulin mu
Anti-HBs: Antibody Hepatitis B surface
APRI score: Aspartate and platelets ratio index score
AST: Aspartate aminotrasnferase
CBC: Complete Blood Count
cccDNA: covalently closed circular DNA
CDC: Centre for Disease Control
CHB: Chronic hepatitis B
CI: Confidence interval
CMV: Cytomegalovirus
DNA: Deoxyribonucleic acid
HbcAg: Hepatitis B core antigen
HbeAg: Hepatitis B e antigen
HBIG: Hepatitis B immune globulin
HbsAb: Hepatitis B surface antibody
HBsAg: Hepatitis B surface antigen
HBV: Hepatitis B Virus
HbxAg: Hepatitis B Virus X antigen
HCC: Hepatocellular Carcinoma
HCV: Hepatitis C Virus
HCW’s: Health Care Workers.
